# Effect of Different CH_3_NH_3_PbI_3_ Morphologies on Photovoltaic Properties of Perovskite Solar Cells

**DOI:** 10.1186/s11671-018-2556-8

**Published:** 2018-05-08

**Authors:** Lung-Chien Chen, Kuan-Lin Lee, Wen-Ti Wu, Chien-Feng Hsu, Zong-Liang Tseng, Xiao Hong Sun, Yu-Ting Kao

**Affiliations:** 1 Department of Electro-Optical Engineering, National Taipei University of Technology, 1, Section 3, Chung-Hsiao E. Road, Taipei, 106 Taiwan; 2 Institute of Chemistry, Academia Sinica, 128, Sec. 2, Academia Rd., Nankang, Taipei, 115 Taiwan; 3 Henan Key Lab of Laser and Opto-electric Information Technology, School of Information Engineering, Zhengzhou University, Science Road 100, Zhengzhou, Henan China

**Keywords:** Solar cells, Perovskite, CH_3_NH_3_PbI_3_, Two-step deposition

## Abstract

In this study, the perovskite layers were prepared by two-step wet process with different CH_3_NH_3_I (MAI) concentrations. The cell structure was glass/FTO/TiO_2_-mesoporous/CH_3_NH_3_PbI_3_ (MAPbI_3_)/spiro-OMeTAD/Ag. The MAPbI_3_ perovskite films were prepared using high and low MAI concentrations in a two-step process. The perovskite films were optimized at different spin coating speed and different annealing temperatures to enhance the power conversion efficiency (PCE) of perovskite solar cells. The PCE of the resulting device based on the different perovskite morphologies was discussed. The PCE of the best cell was up to 17.42%, open circuit voltage of 0.97 V, short current density of 24.06 mA/cm^2^, and fill factor of 0.747.

## Background

Organic perovskite films have drawn much attention for better power conversion efficiency in thin film-type solar cells [1–3]. Many growth methods have been developed to prepare perovskite films. Among them, a two-step method is widely used due to its high film quality and reliability of the resulting films [4, 5]. The perovskite is a versatile material prepared from abundant and low-cost compounds, also having unique optical and long excitonic properties, as well as good electrical conductivity. The power conversion efficiency (PCE) of perovskite solar cells has been improved from 3.8 to 22.1% in recent years.

There are two methods for preparing perovskite films: one-step and two-step methods for CH_3_NH_3_PbI_3_ films; the one-step method is that the PbI_2_ and CH_3_NH_3_I (MAI) are mixed in a solvent to form CH_3_NH_3_PbI_3_ films, such as vacuum flash-assisted solution processes, [5] solvent engineering, [6] humidity control, [7, 8] and mixed precursors [9]. Although the one-step method is the most widely used method to prepare the perovskite solar cells, it needs to dissolve both the organic and the inorganic precursors, which reduced the control of the film property including thickness, uniformity, and morphology. The two-step method is that the PbI_2_ films were first prepared and subsequently reacted with MAI to form CH_3_NH_3_PbI_3_ films. In 2013, Bi et al. [10] first showed the PCE of 9.5% by using two-step method. They prepare PbI_2_ films on mesoporous TiO_2_ films the by spin coating a PbI_2_ solution in dimethylformamide (DMF). After drying, the films were dipped in a solution of MAI in 2-propanol to form high-quality CH_3_NH_3_PbI_3_ films for the perovskite solar cells. In the same year, Burschka et al. [11] showed the certification for the perovskite solar cells prepared by the two-step method and confirmed a power conversion efficiency of 14.14% measured under standard AM1.5G reporting conditions. After that, many studies using the two-step method to improve the PCE of the perovskite solar cells were reported [12–18]. Moreover, long-term stability is important for the future application of perovskite devices. Several nanostructures, like carbon layer [19] and graphene oxide-modified PEDOT:PSS [20], have been used to suppress degradation in the device and improve their performance. However, few studies discuss the effect of different surface morphology on photovoltaic properties of perovskite solar cells.

In this study, we controlled the grain size and morphology of CH_3_NH_3_PbI_3_ by different MAI concentration, annealing, and two-step. Moreover, it was found that the surface morphology of CH_3_NH_3_PbI_3_ films using low MAI concentrations showed large perovskite grains, but the morphology of CH_3_NH_3_PbI_3_ films using high MAI concentrations showed dense and smooth grains. Photovoltaic conversion efficiency of the resulting cells based on the different perovskite morphologies was analyzed using XRD spectra, SEM, UV-vis absorption spectroscopy, and photoluminescence (PL) spectra. As a result, the power conversion efficiency of the best cell was up to 17.42%.

## Methods

**Fig. 1:**
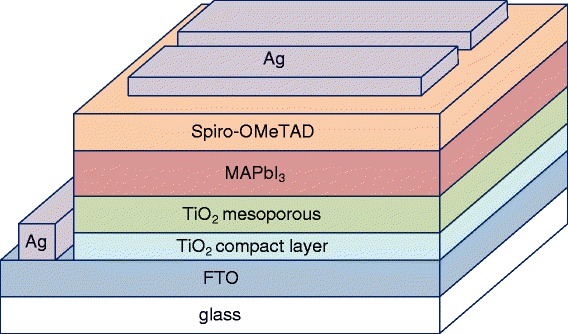
Schematic of the complete structure

In this study, fluorine-doped tin oxide (FTO) glass as substrate was cut into small pieces with a size of 1.5 × 1.5 cm^2^. The FTO glass substrates were thoroughly cleaned with acetone, ethanol, and deionized (DI) water in an ultrasonic oscillator for 5 min, respectively, and dried with nitrogen. A 50-nm compact TiO_2_ blocking layer was first deposited onto the surface of the precleaned FTO substrate by spray pyrolysis method at a temperature of 500 °C, using a solution of 0.2 M Ti-isopropoxide and 2 M acetylacetone in isopropanol. The mesoporous layer TiO_2_ was deposited by spin coating a diluted paste (Dyesol 18NR-T), followed by heating to 450 °C. Next, the two-step method was employed to deposit a perovskite layer. PbI_2_ (Alfa Aesar, 99.9985% purity) was deposited via spin coating from a solution 1 mol/L PbI_2_ in dimethylformamide (DMF) that was heated to 70 °C, with a spin coating speed of 7000 rpm. MAPbI_3_ was formed by dipping the slide into a 10-mg/mL MAII in isopropanol (IPA) solution with different concentrations for 30 s. After removing the excess IPA, the perovskite films were then placed on a hot plate set at 100 °C for 20 min. The composition of hole transport material was 0.170 M 2,2′,7,7′-tetrakis(*N*,*N*-di-p-methoxyphenyl-amine)-9,99-spirobifluorene (spiro-OMeTAD, Lumtec), with the addition of 60 mM bis(trifluoromethane)sulfonimide lithium salt (LiTFSI, 99.95%, Aldrich) and 200 mM 4-tert-butylpyridine (TBP, 99%, Aldrich). The CH_3_NH_3_PbI_3_/TiO_2_ films were coated with a spiro-OMeTAD solution using the spin coating method at 4000 rpm. For the electrical contact, a 100-nm Ag film was deposited onto the solar cell by thermal evaporation. The resultant device was composed of silver/spiro-OMeTAD/MAPbI_3_/TiO_2_ mesoporous layer/TiO_2_ compact layer/FTO/glass. Figure 1 schematically depicts the complete structure. The current density-voltage (J-V) curves of solar cells were obtained using a source measurement unit (Keithly 2400). The photoluminescence spectra of the CN_3_NH_3_PbI_3_/glass samples were measured using a microscope-based spectrometer. The active area of the devices is 2 × 5 mm^2^ by a shadow mask. The X-ray diffraction patterns of the CN_3_NH_3_PbI_3_/glass samples were recorded using a theta-2theta mode.

## Results and Discussion

**Fig. 2:**
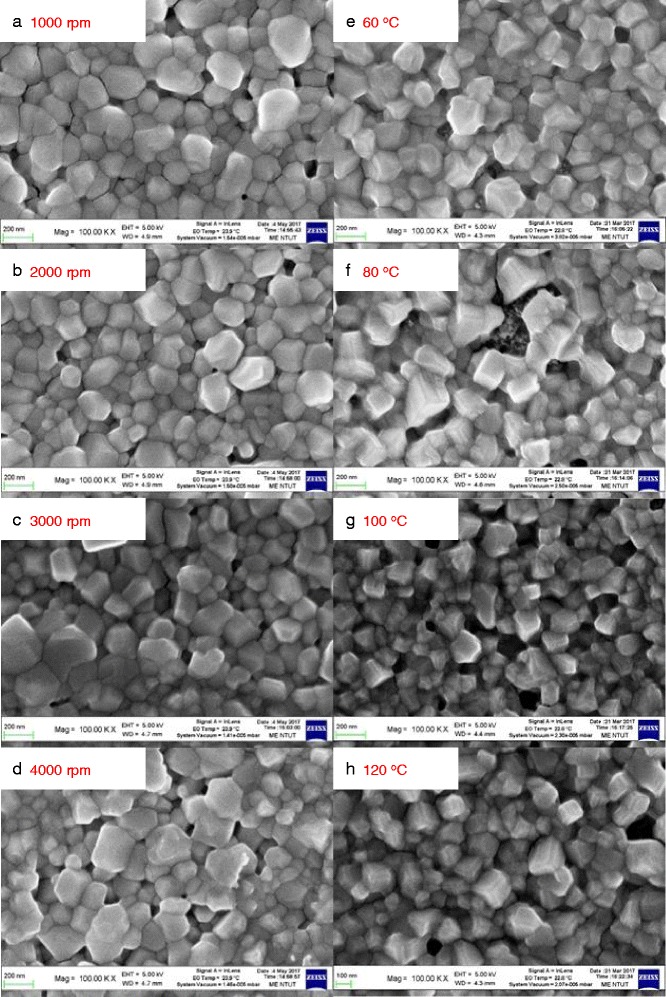
Top-view SEM images of the MAPbI_3_ perovskite films prepared by low-concentration MAI (10 mg/mL) with **a-d** various spin coating speeds and **e-h** annealing treatments

Figure 2 shows the top-view (left column) and cross-sectional (right column) SEM images of the MAPbI_3_ perovskite films prepared by low-concentration MAI (10 mg/mL) and underwent annealing treatment at different temperatures. It was found that there is a large amount of perovskite particles on the surface and have a tetragonal morphology, as shown in Fig. 2a. The particles size and surface morphology of the perovskite films prepared by low-concentration MAI are similar for all samples.

**Fig. 3:**
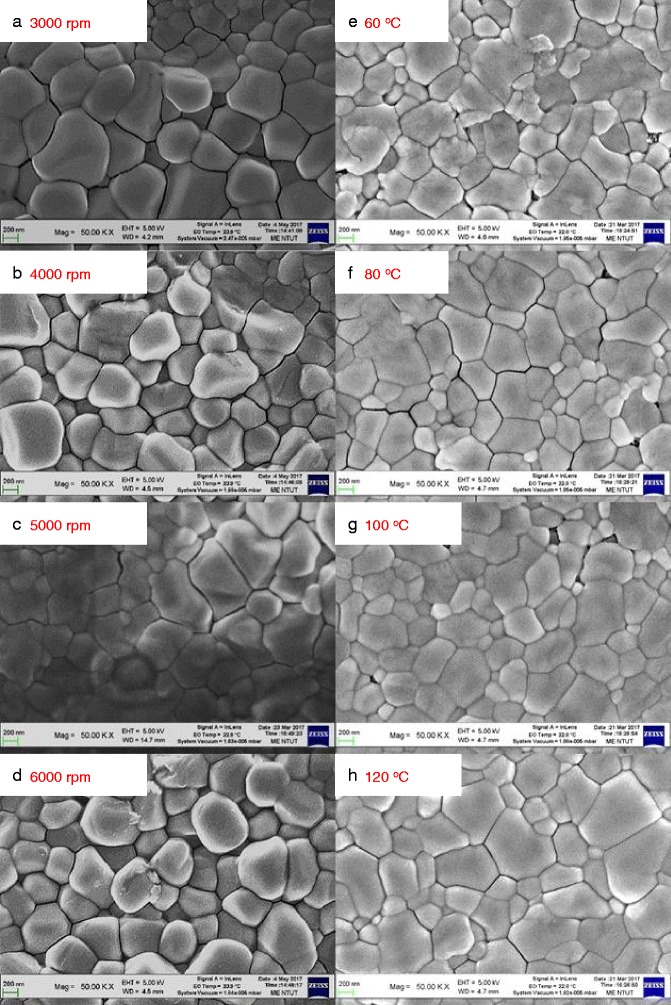
Top-view SEM images of the MAPbI_3_ perovskite films prepared by high-concentration MAI (10 mg/mL) with **a-d** various spin coating speeds and **e-h** annealing treatments

Figure 3 shows the top-view (left column) and cross-sectional (right column) SEM images of the MAPbI_3_ perovskite films prepared by high-concentration MAI (40 mg/mL) and underwent annealing treatment at different temperatures. Perovskite prepared by high-concentration MAI shows tetragonal crystals, the average MAPbI_3_ domain size from about 200 nm to about 600 nm, as shown in Fig. 3. The morphology is different to that of the perovskite prepared by low-concentration MAI. It was found that there are some PbI_2_ grains on the surface of the MAPbI_3_ perovskite film with 60 °C annealing. Those are residues caused by the incomplete reaction. The domain size and surface morphology of the perovskite films prepared by high-concentration MAI are similar for all samples.

**Fig. 4:**
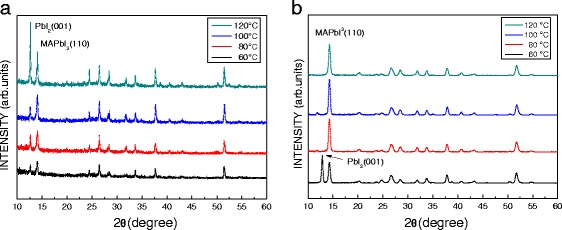
XRD patterns of the MAPbI_3_ films with **a** low- and **b** high-concentration MAI

Figure 4 shows the XRD patterns of the MAPbI_3_ films prepared by (a) low- and (b) high-concentration MAI with different annealing temperatures. As shown in Fig. 4a, two main diffraction peaks are observed at 12.6° and 14.4°, corresponding to PbI_2_ (001) and MAPbI_3_ (110) phases, respectively. The intensity of the PbI_2_ (001) peak is higher than that of the MAPbI_3_ (110) peak when the annealing temperature of the MAPbI_3_ film increases up to 120 °C. The MAPbI_3_ film is decomposed into a bi-phase film of the MAI and PbI_2_, leading to poor efficiency of the perovskite solar cells. Similarly, as shown in Fig. 4b, as the annealing temperature is 60 °C, two main diffraction peaks are observed at 12.8° and 14.3°, corresponding to PbI_2_ (001) and MAPbI_3_ (110) phases, respectively. However, the single peak corresponding to the MAPbI_3_ (110) phase is observed when the annealing temperature of the MAPbI_3_ film increases up to over 80 °C. The MAI and PbI_2_ are formed into the MAPbI_3_ film, completely.

**Fig. 5:**
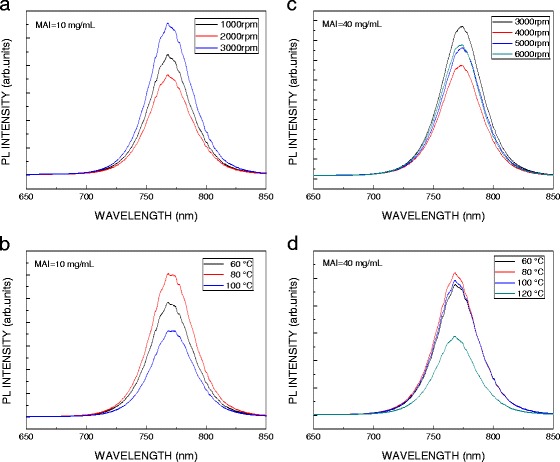
PL spectra of MAPbI_3_ prepared by low-concentration MAI with **a** various spin coating speeds and **b** annealing temperatures and prepared by high-concentration MAI with **c** various spin coating speeds and **d** annealing temperatures

The intensity of photoluminescence (PL) spectrum is related to the lifetime of an exciton in the perovskite film and in the interface between TiO_2_ and perovskite films. The lifetime of the exciton is longer, and the intensity of PL spectrum is stronger; the decomposition rate of an exciton in the interface between TiO_2_ and perovskite films is faster, and the intensity of PL spectrum is weaker. Figure 5 plots PL spectra of the MAPbI_3_ prepared by low- and high-concentration MAI with various spin coating speeds and annealing temperatures. As shown in Fig. 5a, b, the optimum spin coating speed and annealing temperature for the MAPbI_3_ prepared by low-concentration MAI are 2000 rpm and 100 °C, respectively. On the other hand, as shown in Fig. 5c, d, the optimum spin coating speed and annealing temperature for the MAPbI_3_ prepared by high-concentration MAI are 4000 rpm and 120 °C, respectively.

**Fig. 6:**
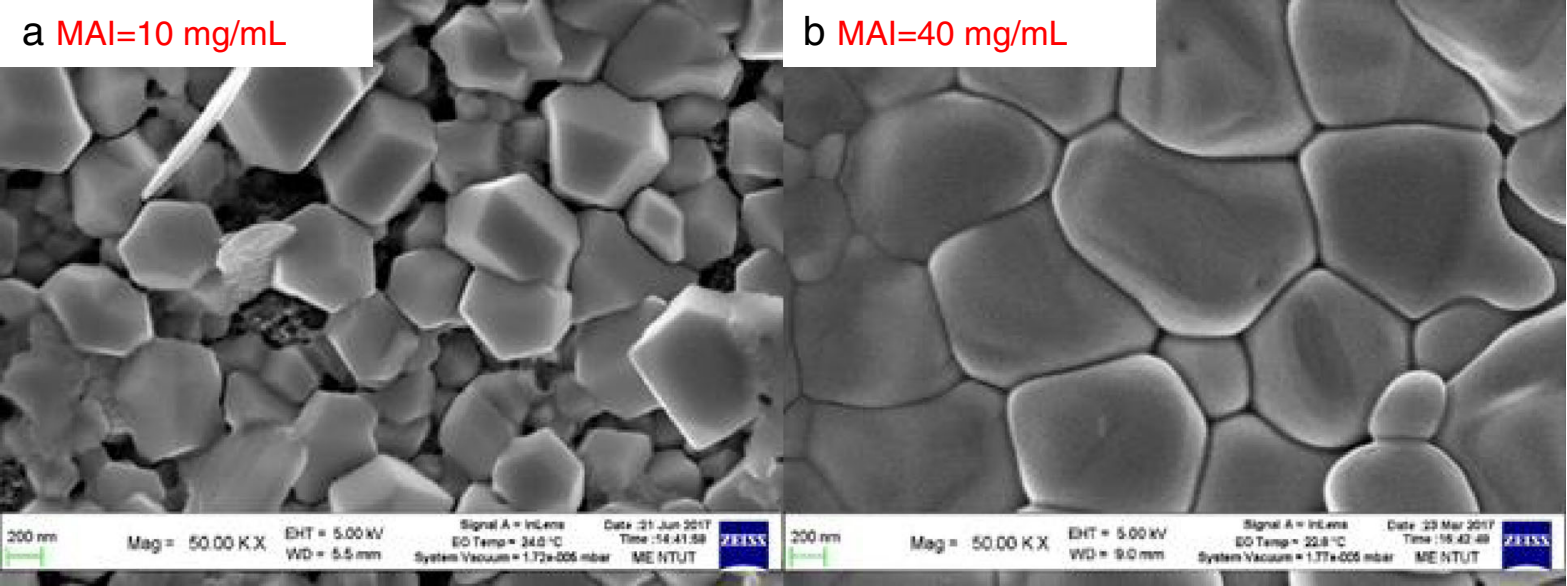
SEM images of the MAPbI_3_ perovskite films with **a** low- and **b** high-concentration MAI solution under optimum conditions, respectively

Figure 6 shows the SEM images of the MAPbI_3_ perovskite films with low- and high-concentration MAI solution under optimum conditions, respectively. The surface morphology of the MAPbI_3_ perovskite films with low-concentration MAI is rougher than that of the MAPbI_3_ perovskite films with high-concentration MAI. The grain of the latter is compact and smooth. Also, the coverage rate of the surface of the latter is better than that of the former.

**Fig. 7:**
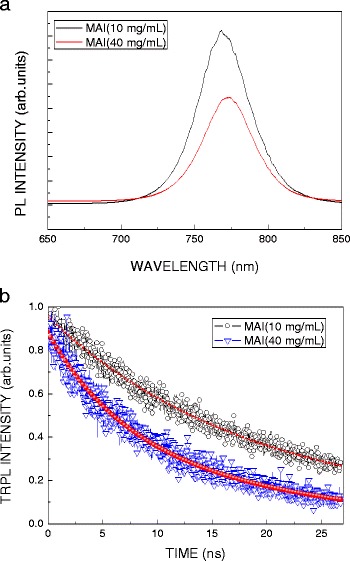
**a** PL and **b** TRPL spectra of MAPbI_3_ films with different concentrations

Figure 7a shows the PL spectra of the MAPbI_3_ films with different MAI concentrations. The peak position of PL spectrum increases from 768 to 773 nm when the MAI concentration increases from 10 to 40 mg/mL. The redshift might be associated with the reaction of PbI_2_ and MAI [21]. As the PbI_2_ film is reacted with the MAI solution and formed the MAPbI_3_ perovskite film, the band gap is shifted toward 1.55 eV. Also, the intensity of PL spectrum of the MAPbI_3_ perovskite film using high MAI concentration is decay. To explore the original cause, time-resolved photoluminescence (TRPL) was employed to study the lifetime of the excitons. Therefore, the excitons can be quickly extracted to the FTO substrate, for the MAPbI_3_ perovskite film using high MAI concentration. According to the TRPL spectra shown in Fig. 7b, the lifetime of the MAI perovskite films prepared by low and high concentration is 25 and 14 ns, respectively. It can clearly be seen that the exciton lifetime of the MAI perovskite films prepared by high MAI concentration is relatively short, which can be used to explain why the decomposition rate of the excitons is faster. The interface between of TiO_2_ and perovskite prepared by high MAI concentration is smooth, such that the excitons are separated and extracted quickly to the FTO substrate, as shown in Fig. 7b. In addition, it is possible to improve the film quality, resulting in an increase in the speed of the electrons decomposition.

**Fig. 8:**
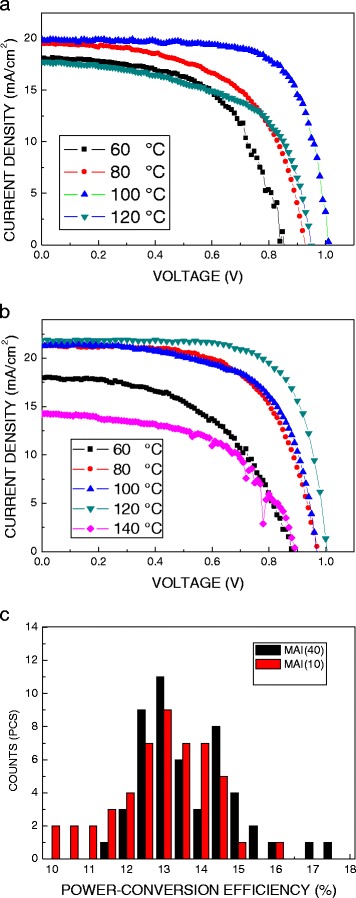
J-V curves of the perovskite solar cells prepared using **a** low-concentration MAI and **b** high-concentration MAI with different annealing temperatures. **c** Histograms of PCE of the perovskite solar cells prepared by high-concentration MAI under the optimum process condition for 50 devices

Figure 8a, b plots the J-V curves of the perovskite solar cells prepared by low- and high-concentration MAI with different annealing temperatures, respectively. To compare the short-circuit current density Jsc, the perovskite solar cells prepared by high-concentration MAI are higher around 2 mA/cm^2^ than that of the cells prepared by low-concentration MAI. This may be contributed by better quality of the perovskite films prepared by high-concentration MAI, such that it has a higher absorbance, resulting in a higher photocurrent. Besides, the charge transfer resistance in the perovskite films prepared by high-concentration MAI is small due to the smooth morphology. The films with the smooth morphology can not only increase the contact area between perovskite film and spiro-MeTAD film but also enhance the photoelectric conversion efficiency of solar cells [22, 23]. On the other hand, the cells prepared by low-concentration MAI show high Voc. It may be caused by PbI_2_ residues in the perovskite thin film [22, 23]. To check the reproducibility of performance, power conversion efficiency (PCE) is compared using histograms obtained from 50 perovskite devices prepared by low- and high-concentration MAI, as shown in Fig. 8c. As can be seen from the results, the devices performed extremely well. The average PCE of the perovskite solar cells prepared by low- and high-concentration MAI is 13 and 13.7% with a standard deviation of 1.293 and 1.275%, respectively. As shown in Fig. 8c, more than 75% of the cells show PCE above 13% under one sun conditions, for the perovskite solar cells prepared by high-concentration MAI. That indicates good reproducibility. The optimum results show the power conversion efficiency of 17.42%, open-circuit voltage of 0.97 V, current density of 24.06 mA/cm^2^, and fill factor of 0.747.

## Conclusions

In this study, the perovskite films prepared by high-concentration MAI were used to form solar cells. The effects of different morphologies of the films on the solar cells were investigated. The J-V characteristic curve of perovskite solar cells was used to improve the photoelectric conversion efficiency. The results show that the power conversion efficiency was up to 17.42%, open circuit voltage of 0.97 V, current density of 24.06 mA/cm^2^, and fill factor of 74.66% was the best characteristic.

## Abbreviations

FTO: Fluorine-doped tin oxide
J-V: Current density-voltage
MAI: CH_3_NH_3_I
MAPbI_3_: CH_3_NH_3_PbI_3_
PCE: Power conversion efficiency
PL: Photoluminescence
SEM: Scanning electron microscope
TRPL: Time-resolved photoluminescence
XRD: X-ray diffractometer
